# Thermal Unfolding Pathway of PHD2 Catalytic Domain in Three Different PHD2 Species: Computational Approaches

**DOI:** 10.1371/journal.pone.0047061

**Published:** 2012-10-15

**Authors:** Hamid Hadi-Alijanvand, Elizabeth A. Proctor, Bahram Goliaei, Nikolay V. Dokholyan, Ali A. Moosavi-Movahedi

**Affiliations:** 1 Institute of Biochemistry and Biophysics, University of Tehran, Tehran, Iran; 2 Program in Molecular and Cellular Biophysics, University of North Carolina at Chapel Hill, Chapel Hill, North Carolina, United States of America; 3 Department of Biochemistry and Biophysics, School of Medicine, University of North Carolina at Chapel Hill, Chapel Hill, North Carolina, United States of America; 4 Curriculum in Bioinformatics and Computational Biology, University of North Carolina at Chapel Hill, Chapel Hill, North Carolina, United States of America; 5 Center of Excellence in Biothermodynamics, Institute of Biochemistry and Biophysics, University of Tehran, Tehran, Iran; University of South Florida College of Medicine, United States of America

## Abstract

Prolyl hydroxylase domain 2 containing protein (PHD2) is a key protein in regulation of angiogenesis and metastasis. In normoxic condition, PHD2 triggers the degradation of hypoxia-inducible factor 1 (HIF-1α) that induces the expression of hypoxia response genes. Therefore the correct function of PHD2 would inhibit angiogenesis and consequent metastasis of tumor cells in normoxic condition. PHD2 mutations were reported in some common cancers. However, high levels of HIF-1α protein were observed even in normoxic metastatic tumors with normal expression of wild type PHD2. PHD2 malfunctions due to protein misfolding may be the underlying reason of metastasis and invasion in such cases. In this study, we scrutinize the unfolding pathways of the PHD2 catalytic domain’s possible species and demonstrate the properties of their unfolding states by computational approaches. Our study introduces the possibility of aggregation disaster for the prominent species of PHD2 during its partial unfolding. This may justify PHD2 inability to regulate HIF-1α level in some normoxic tumor types.

## Introduction

Eukaryotic cells have an arsenal of genes expressed in response to hypoxia [1]. Regulation of hypoxia response genes plays a significant role in cell survival in hypoxic conditions like high altitude, strokes and asthma. These genes include sets that induce processes such as angiogenesis, cell motility and glucose uptake. Hypoxia inducible factor 1 (HIF-1) is a transcription factor that orchestrates the expression of hypoxia response genes [2], [3]. The stability of HIF-1α (the regulatory domain of HIF-1) is mainly determined by prolyl hydroxylase domain containing protein 2 (PHD2). In normoxic condition, PHD2 performs HIF-1α hydroxylation and triggers its degradation. When oxygen pressure is low, PHD2 can not start HIF-1α degradation so HIF-1α level remains high and hypoxia response genes are induced. PHD2 catalytic domain has jelly roll architecture with a double stranded beta helix structure composed of eight strands. Fe (II) is necessary for the PHD2 function and it coordinates to residues that reside in active site lumen [4]–[6].

Partial unfolding of protein structure is a prerequisite for protein misfolding and aggregation. Upon protein unfolding, the structure of unfolded protein suffers various disturbances that competent protein structure for aggregation disaster [7]. The correlation between beta strand propensity and protein aggregation was demonstrated [8]. The naturally beta sheet proteins have many mechanisms to suppress beta strand stimulated protein aggregation [9]. But such inherently inhibitory mechanisms of aggregation fade upon protein denaturation. Protein hydration is another critical factor for protein stability. The small value of globular protein solvation energy indicates high accessibility of polar residues. Partial protein unfolding may causes high solvent exposure of hydrophobic residues therefore the increment of hydrophobic accessible surface area makes sticky regions on protein surface then it prepares conditions for protein self-assembly [10]. While there are some methods to measure the amount of protein exposed region’s hydrophobicity, solvation energy measurement is a convenient method. Another protein aggregation’s risk factor is the presence of metal ions in protein structures. There are proofs that indicate unnatural quantity of iron and zinc atoms in tissues with high amount of aggregated proteins [11]. It is possible that these ions accelerate primary stages of protein aggregation after partial protein unfolding or even at first stage of protein folding. In this study, we try to find possible risk factors that accelerate PHD2 protein misfolding upon partial unfolding.

**Figure 1:**
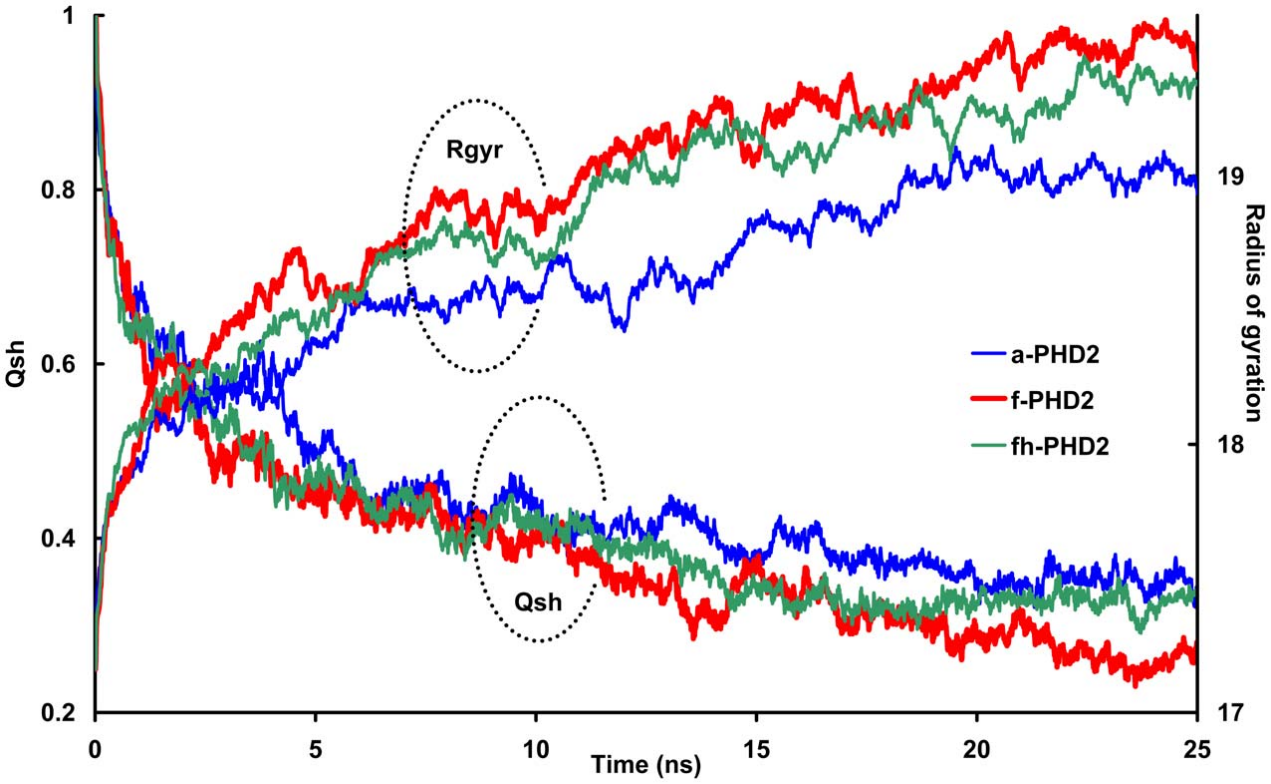
Representation of unfolding process metrics. The fluctuations of long range native contact fraction (*Qsh*) and radius of gyration as a function of simulation time are represented.

Studies have indicated that PHD2 silencing was concomitant with cancer cell survival and metastasis [12], [13]. It has been reported that PHD2 gene carries mutations in some cancers. Such mutations may cause PHD2 inactivation and by this way promotes HIF-1α stabilization, angiogenesis and metastasis [14], [15]. However, many groups have reported a high level of HIF-1α in some cancer types even in normoxic condition and in spite of high level of wild type PHD2 protein [16], [17]. Such observations improve the possibility of PHD2 misfolding issues such as instability and aggregation. For investigating the possible misfolding of PHD2, it is necessary to illustrate the PHD2 unfolding pathway and characterize its possible unfolding states. A common computational method to study protein unfolding pathway is heating of the protein system to temperatures higher than the protein transition point [18]. Molecular dynamic simulation helps us to study the unfolding phenomena in atomic details [19], [20].

**Figure 2:**
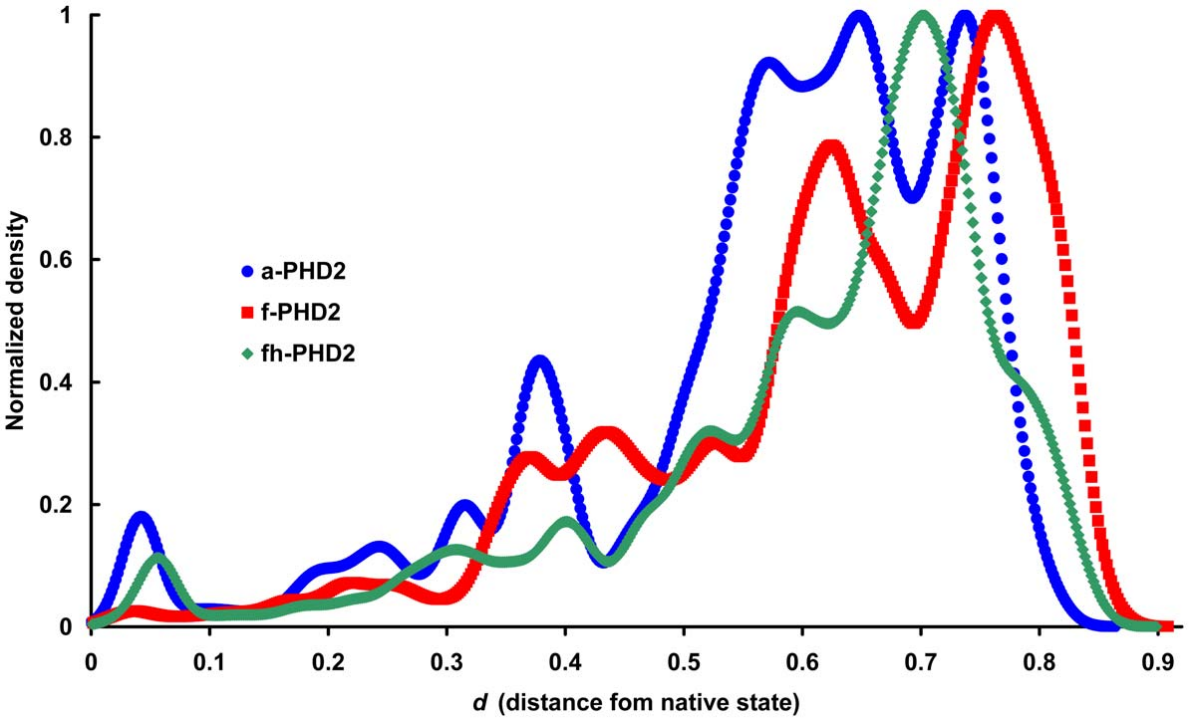
The distributions of d metric as a reaction coordinate upon thermal unfolding. The normalized density of *d* as reaction coordinate is depicted for PHD2 species.

There is no report about the PHD2 unfolding pathway. We explain thermal unfolding pathway of the PHD2 catalytic domain in three different kinds of PHD2 via computational approaches. Three PHD2 species that are studied here include: the nascent PHD2 without Fe (II) ion (a-PHD2); the mature PHD2 that has an iron in its active site (f-PHD2); and f-PHD2 with protonated histidine residues (fh-PHD2) which may appear in acidic compartments of cells. The study of PHD2 species unfolding pathways and characterization of their unfolding states may shed light on the possible reasons of PHD2 malfunction in some normoxic cancer cells.

## Methods

We extracted the initial structure of PHD2 from the crystal structure (PDB ID: 3HQU chain A). All atom molecular dynamic (MD) simulations were carried out using NAMD 2.8 software [21] and CHARMM22 protein force filed with CMAP corrections [22]. Generalized Born implicit solvent was used to accelerate simulations. The non-bonded interactions cutoff and ion concentration were set to 16 Å and 0.15 M respectively. The MD time step was set to 1.0 femtosecond. After 0.50 nanosecond (ns) minimization, protein was heated from 0 to 500 K gradually. Then protein structure was equilibrated for 25 ns at 500 K with langevin thermostat. The interaction energy between different PHD2 structural elements is computed via NAMD energy tool of VMD.

**Figure 3:**
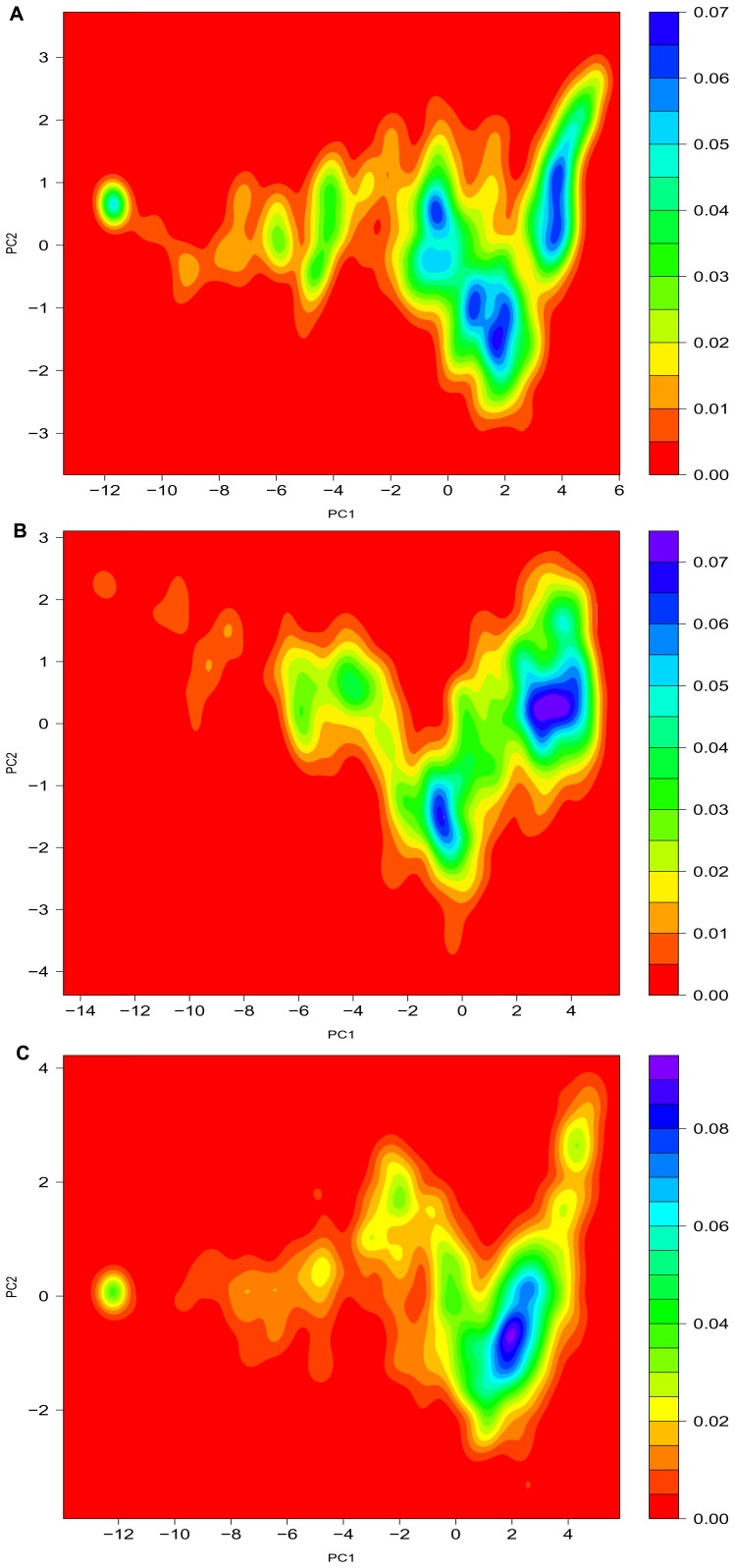
2D kernel densities of PCA for the PHD2 species unfolding states. The most fold state resides in left side of panels and the most unfold states reside in right side of panels. The densities of a-PHD2, f-PHD2 and fh-PHD2 are depicted in panel A, B and C respectively. The panels are the 2D kernel density maps for the principal components 1, 2. Principal components are computed by utilizing the properties space used to build the *d* metric. Horizontal and vertical dimensions indicate first and second principal components respectively. The contour levels indicate the density. Darker region is the most populated region.

**Figure 4:**
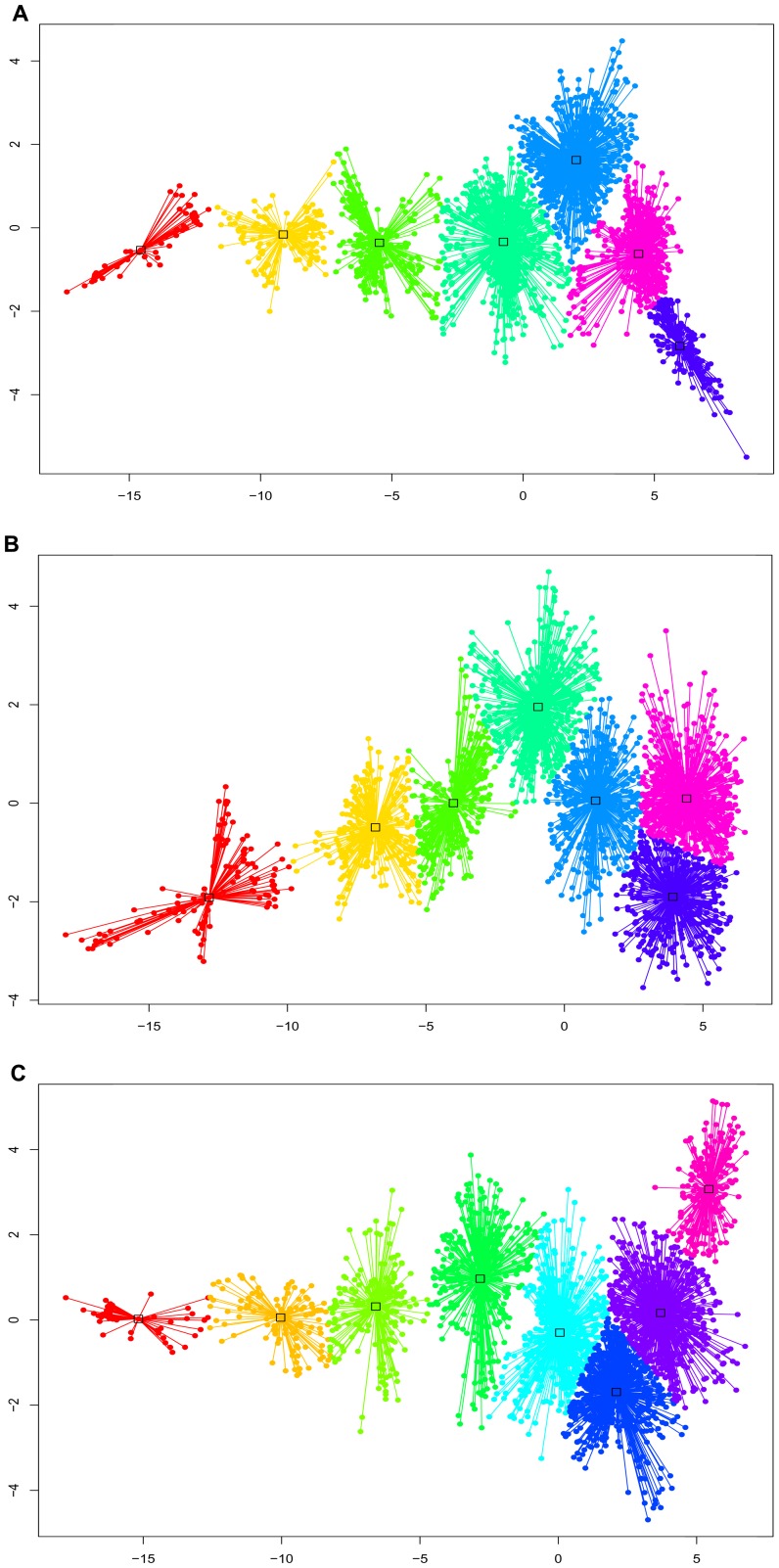
The results of structures clustering by Affinity propagation (AP) method for PHD2 species. The most fold cluster resides in left side of panels and the most unfold clusters reside in right side of panels. The structures’ clusters of a-PHD2, f-PHD2 and fh-PHD2 are depicted in panel A, B and C respectively. The panels are the results of affinity propagation clustering method. After reducing the dimensionality of the properties space used to build the *d* metric by n-MDS, AP clustering is performed. X and Y axes are the output vectors of n-MDS. Points represent structures in AP method. Clusters (states) are declared with different colors.

The parameter *Qsh* measures the fraction of long-range native contacts between C_α_ atoms with sequence separation of at least 7 residues and 3D separation <10 Å. This metric is inferred from [23].
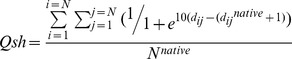



Where *N* is the length of protein, *d_ij_* denotes the distance between the contacting residues in the sample structure, *d^native^* denotes the distance between these same residues in the native reference structure, and *N^native^* is the total number of contacts in the native structure.

**Figure 5:**
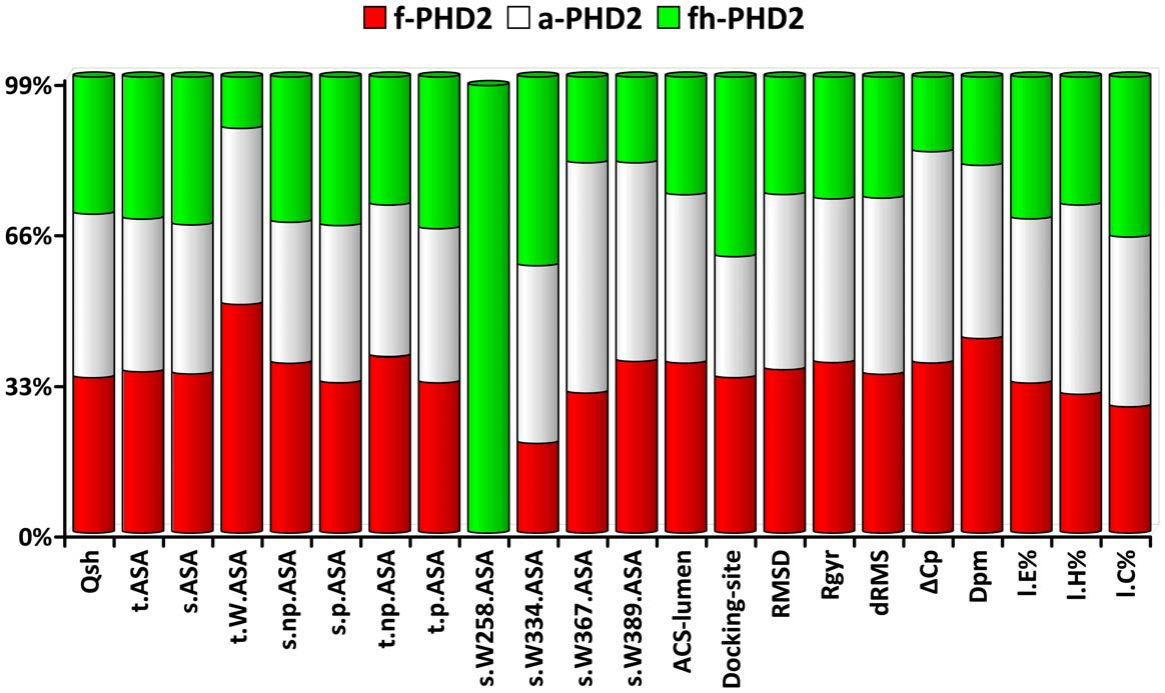
Comparing the percentage of PHD2 species contribution to each variable during partial unfolding. The contribution of PHD2 species to each property’s information content is represented in percent. p, np note polar or non-polar parts of the molecule. ASA stands for accessible surface area, t and s stand for total and side chain respectively. Rgyr stands for radius of gyration. Dpm notes the dipole moment. W stands for tryptophan residue. Docking site and active site lumen are the ASA of corresponding parts of PHD2. l.E% and l.H% stand for the amount of strand or helix structure melting respectively. l.C% notes the amount of appeared coiled structure.

We wrote Tcl script to compute accessible surface area, radius of gyration, secondary structure content and RMSD of structures along trajectories by using VMD 1.9.1 [24]. After removal of rotation and translation of structures, pairwise C_α_ RMSDs were computed. Pairwise C_α_ RMSDs were used to guess PHD2 species stability. We calculated the dRMS as a criterion for the deviation of the distance matrix between same C_α_ pairs in the native and query structures [25].

In order to evaluate the difference of properties between query structures and the native state, we computed “*d*” metric based on Daggett method [26]. It has been assumed that *d* metric acts as an unfolding reaction coordinate.

**Table 1:** The changes of protein solvation free energy upon unfolding.

	*d*	Polar	Apolar	Total
a-PHD2	0.2–0.4	−71	68	−3
	0.4–0.6	−42	112	69
	0.6–0.8	−275	177	−98
	0.8–1.0	−4	176	172
f-PHD2	0.2–0.4	51	85	136
	0.4–0.6	4	109	113
	0.6–0.8	123	110	232
	0.8–1.0	246	133	379
fh-PHD2	0.2–0.4	−160	92	−68
	0.4–0.6	−153	122	−31
	0.6–0.8	−325	188	−137
	0.8–1.0	−230	198	−32

For each state with a special *d* value, we compute the free energy of solvation for the state’s representative structure. The difference between this value and solvation free energy of native state (*d* = 0.0) is reported here in kcal/mol.

**Figure 6:**
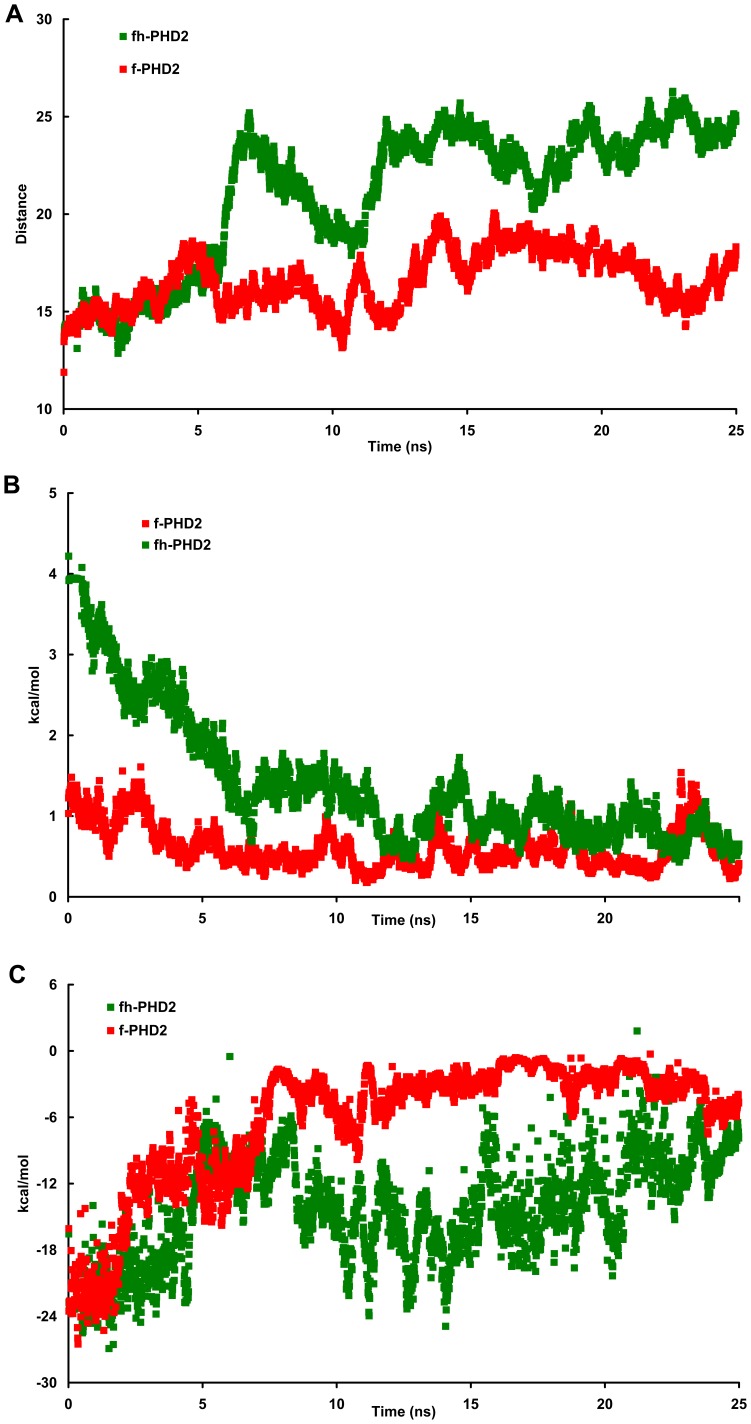
The consequences of Fe atom detaching from PHD2 active site lumen. The distance between Fe ion and arginine 383 is computed during f and fh-PHD2 unfolding (A). The interaction energy (repulsion) between Fe atom and f or fh-PHD2 basic residues is computed (B). The interaction energy between strand HD (residues 308 to 320) and strand H (residues 372 to 377) is computed for f and fh-PHD2 (C).

**Figure 7:**
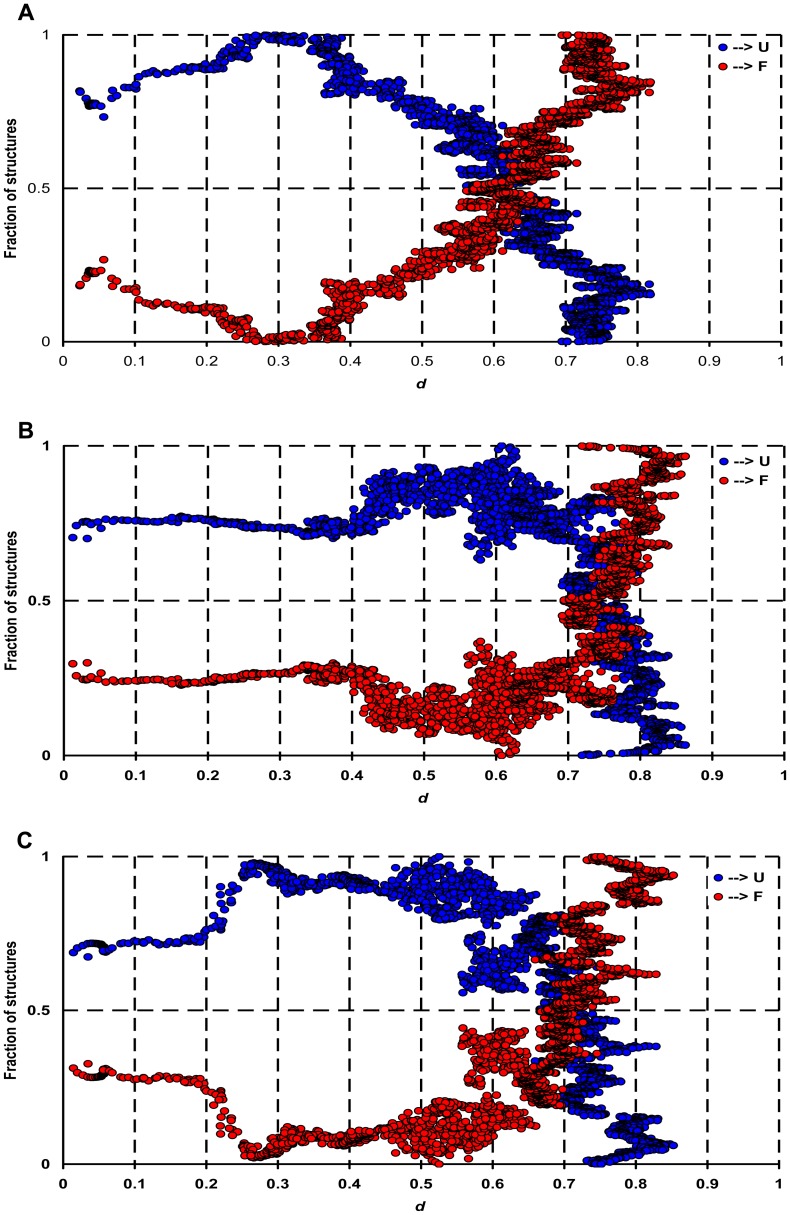
The maximal stability of PHD2 species. The fraction of structures which are more folded (red spheres) or more denatured (blue spheres) than the corresponding structure of each *d* value is represented. The junction of blue and red spheres indicates the position of main transition for (A) a-PHD2, (B) f-PHD2 and (C) fh-PHD2.

The multidimensional property space used in this study to construct the *d* metric includes total or side chain solvent accessible surface area (t/s ASA), ASA for total and side chain region of polar (p) and non-polar (np) residues, ASA of all tryptophan (W) residues, side chain ASA of each tryptophan residue of PHD2 (W258, W334, W367 and W389), ASA of active site lumen (ACS-lumen) and docking site region (Docking-site) of PHD2 structure [4], *Qsh* metric, RMSD and dRMS relative to crystal structure, radius of gyration, dipole moment for each frame, changes in heat capacity (ΔCp) computed based on the changes in polar and apolar ASA relative to native structure [27], the percent of melted helixes (l.H) and strands (l.E) and the percent of appeared coil regions (l.C).

We calculated the normalized fraction of unfolding in regard to crystal structure for each property during simulations (data are normalized to the highest value). By composing the normalized fractions of unfolding based on Daggett method [26], we compute the *d* metric for each simulation step as follow;
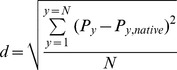



Where *N* is the number of properties, *P* denotes the normalized fraction of unfolding based on each property (*y*). The minimization steps’ structures were considered as the native structures’ pool. For the pool of native structures, the average of normalized fractions of unfolding was computed based on *y* and called *P_y,native_*.

Based on each property (e.g. t.ASA) we calculated the fraction of protein unfolding for each unfolding state (*us*). We introduced a Shannon entropy by assuming the equal unfolding fraction for all states (*f_eU_*). Another Shannon entropy was calculated based on the observed unfolding fractions of states (*f_oU_*). The information content (*I*) of the desired property was computed as follow [28]:
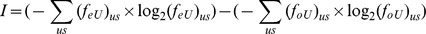



We computed 2D kernel densities and performed affinity propagation clustering (AP), principal component analysis (PCA) and non-metric multidimensional scaling (n-MDS) by using the R 1.14 software [29]–[31]. The solvation free energies of polar and apolar parts of structures were computed by APBS 1.3 software [32].

## Results and Discussion

The analysis of thermal unfolding MD trajectories provides insight to reveal the key phenomena of the first steps of protein unfolding. The study of structural aspects of states along the unfolding pathway may reveal the basis of wild type PHD2 malfunction in some types of tumors.

Variations in protein dimension and native contacts are indicators of protein structural changes during thermal denaturation. We compute the radius of gyration and the fraction of long-range native contacts for the structures of PHD2 species along 25 ns thermal unfolding simulations (Figure 1). The corruption of long-range native contacts is apparent by the decrease in *Qsh* value for the a-PHD2 (nascent form of PHD2), f-PHD2 (PHD2 with iron in active site) and fh-PHD2 (f-PHD2 with protonated histidine residues) structures upon heating. Also, an increase in protein radius of gyration indicates that the structures swell as a consequence of partial thermal unfolding. Changes in these metrics indicate that, despite identical protein sequence and initial structure, these three different species of PHD2 exhibit different properties upon unfolding.

A suitable reaction coordinate is necessary to study the unfolding pathway chronologically. The average distance of query structure’s properties from the native state properties (*d*) is a suitable reaction coordinate to analyze protein unfolding [26]. We measure the distance (*d*) of PHD2 unfolded structures from the crystal structure. This metric assigns a value of zero to native structure and one to the most unfolded structures. Probability densities of *d* indicate that, while most structures are a large distance from the native state and therefore unfolded, the distributions of populated structures vary between the different PHD2 species (Figure 2 and Tables S1, S2, S3). This finding implies the possibility of various unfolding pathways for PHD2. For example, f-PHD2 has a clear population when it loses 45% of its similarity to its native state properties (*d* = 0.45) while other species do not have such population. On the other hand, the most populated structure of fh-PHD2 appears at *d* = 0.7 and other two species have the most populated structures in other regions of reaction coordinate.

To confirm the distance (*d*) distributions of structure populations, we perform PCA with the properties used to construct *d* as the reaction coordinate. PCA reduces the multidimensionality of property space [26]. The first two components of PCA cover over 95% of data variance. The 2D kernel density maps of principal components 1 and 2 indicate different highly populated regions for partial unfolding process of PHD2 species (Figure 3). Because the 2D kernel density of PCA components is a population density (X_i_), it is possible to consider it as 2D potential of mean force (PMF) map for unfolding trajectories (PMF = −RTln(X_i_)).

The appearance of highly populated regions in the 2D PCA maps (darker regions) demonstrates that three species of PHD2 have different populated states during thermal unfolding, implying a multi-state model of unfolding. The populated regions of such maps are in good consistency with *d* kernel density distributions (Figure 2). The 2D PCA map for a-PHD2 indicates this species has four populated states in the most unfolded region of its trajectory (Figure 3A). The coordination of Fe (II) to the active site lumen of a-PHD2 creates the f-PHD2 species, decreasing the number of states to two. Protonation of histidine residues in f-PHD2 (fh-PHD2) reduces its unfolded populations to a single populated state (Figure 3C).

What are the properties of the thermal unfolding states of the various PHD2 species? How do the addition of iron and protons to the PHD2 structure change the prominent states of thermal unfolding? In the following section we attempt to answer these questions and reveal the consequences of PHD2 species partial unfolding.

Notably, PCA presumes a linear relationship between variables, and thus ignores non-linear interrelationships between variables. To compensate for this effect, we perform non-metric multidimensional scaling (n-MDS) for the properties used to construct the *d* metric [33]. We use the first two-fitted n-MDS configuration vectors to cluster trajectory structures into separate populations. Although data compressing methods such as PCA and n-MDS reveal separate populations for PHD2 species unfolding, they are unable to find the border and a representative structure for each population. To provide these data, we utilize exemplar-based affinity propagation (AP) clustering [23]. The AP clustering of n-MDS outputs reveals the same populations as when using the *d* metric distribution and 2D-PCA maps for thermal unfolding of PHD2 species (Figure 4). The representative structures are the centroids of the clusters.

Now, we are able to compute various structural and thermodynamical properties of each state (AP cluster) along the unfolding pathway (Tables S4, S5, S6). To simplify the representation of at least 20 different parameters for 7 states of each PHD2 species, we compute the information content of each averaged parameter for all states of each species. A variable with high information content is likely to be important in the process of unfolding. By comparing the information content of each property between different PHD2 species, we capture which properties are more influenced by the addition of iron or protons to a-PHD2 (Figure 5).

The stand-out variables in information content graph are the accessible surface area of tryptophan residues (s.Wxxx.ASA). Tryptophan 258 (W258), which resides at the entrance of the PHD2 active site lumen, is the first tryptophan residue that reaches its maximal exposure in the a-PHD2 species, which occurs when the protein reaches a state that is only 40% unfolded (*d = *0.4), compared to other tryptophan residues in a-PHD2 that do not become exposed until the last steps of protein unfolding (Table S4). Two tryptophan residues (W334 and W389) located along the active site lumen reach their maximal solvent accessibility when fh-PHD2 becomes 60% unfolded. Tryptophan 389 (W389) reaches its maximal accessibility in f-PHD2 when the protein is in 70% unfolded structure. These observations indicate that the coordination of the Fe (II) ion to the PHD2 active site sensitizes tryptophan 389 to structural changes before the completion of thermal unfolding. The acidic environment in fh-PHD2 induces the selective exposure of tryptophan 334 upon PHD2 partial unfolding. Knowledge of the effects of selective exposure of tryptophan residues as the consequence of iron or proton addition are helpful for designing single molecule fluorescent labels for further experimental study of PHD2 unfolding pathway.

Another parameter whose information content varies between the three PHD2 species is the change in heat capacity (ΔCp) along the unfolding pathway (Figure 5 and Table S4). The ΔCp reaches its maximum value in f-PHD2, a-PHD2, and fh-PHD2 sequentially. This results in the f-PHD2 hydrophobic surface becoming accessible to solvent in the early steps of protein unfolding.

By scrutinizing the decay rate of strand structures (l.E%) (Table S4), we observe that f-PHD2 strand structures are more stable than other PHD2 species, although they reside at the same distance from the native structure. The structure of f-PHD2 retains 50% of its strand structures even though the protein is far from the native state (*d* = 0.8). The amount of hydrophobic surface exposure and the percent of remaining strand structures suggest that the common version of cellular PHD2, f-PHD2, may be vulnerable to aggregation upon partial denaturation [7], [34]. These observations provide misfolding and aggregation as a possible mechanism for wild type PHD2 malfunction in normoxic tumor cells.

For further analysis of the aggregation propensity of the states of the various PHD2 species, we compute changes in the protein hydration free energy (ΔG*(h)*) for the representative structures of three species of PHD2 using the APBS software. This software is able to compute the hydration free energy of the polar and apolar parts of each structure (Table 1). A positive Δ*G(h)* indicates an unfavorable hydration upon unfolding [10]. The a-PHD2 species has an unfavorable total hydration free energy when it passes 50 or 90% of the way along its unfolding pathway. Therefore, considering Δ*G(h)*, a-PHD2 is aggregation prone in most unfolded states. In contrast, fh-PHD2 is hydrated all over the unfolding pathway (Table 1). The functional form of PHD2, f-PHD2, does not hydrate along the unfolding pathway. The change in total hydration free energy, the strand structural content, and the ΔCp suggest that f-PHD2 is the aggregation prone species of PHD2.

The quantity of the polar hydration free energy also indicates an odd behavior of the f-PHD2: that the amounts of polar solvation of the f-PHD2 structures do not facilitate f-PHD2 unfolding (the polar part of Δ*G(h)* is usually negative because polar regions become hydrated easily). It is possible that the iron in the f-PHD2 structure causes gathering of polar regions around some nuclei while allowing hydrophobic regions to become exposed during the protein partial unfolding. Therefore, Fe (II) possibly contributes to f-PHD2 aggregation.

Because the iron atom plays critical role in PHD2 function and also is a risk factor for possible PHD2 aggregation, we study in detail the iron experienced events during PHD2 unfolding.

There is an arginine residue (R383) at the end of PHD2 active site lumen which is essential for substrate binding. The position of R383 does not change severely during PHD2 partial unfolding. The measurement of iron – R383 distance indicates that the distance between iron and R383 is increased suddenly in fh-PHD2 unfolding (Figure 6A) while such distance increment did not observe for f-PHD2 unfolding. Possibly, such Fe atom jump is necessary to alleviate harsh conditions during fh-PHD2 unfolding.

The study of Fe atom – basic residues interactions indicates the repulsion between the fh-PHD2 Fe atom and basic residues decreases and reaches to a minimum during first 5 ns where the distance between Fe atom and active site lumen is increased (Figure 6B, S1). On the other hand, the interaction of Fe atom and acidic residues is attractive for f-PHD2 (Figure S2). There are two important strand structures in PHD2 active site lumen. A strand is made by residues from 308 to 320 (strand HD). This strand carries D315 and H313 residues. These two residues with an additional histidine (H374) which resides in the second important strand (residues 372 to 377, strand H) make PHD2 active sites. The strand H and HD cover the floor of active site. The study of interaction energy between strand H and HD indicates that these two strands interaction energy is decreased gradually during f-PHD2 unfolding. It means the floor of f-PHD2 active site is disrupted upon unfolding while the interaction energy between fh-PHD2 strand H and HD does not change critically (Figure 6C).

We propose the protonation of histidine residues makes additional electrostatic attractions between strand H and HD in fh-PHD2. Also it is concluded the Fe atom is pushed out from fh-PHD2 active site lumen therefore it does not make trouble for active site floor integrity. Such Fe atom pushing out causes severe rupture of f-PHD2 active site floor (Figures S3, S4, S5). These observations denote that D315 traps the Fe atom during PHD2 unfolding. D315 acts as a relay between strand HD and the outside of active site lumen. In this study, it is inferred that D315 is free to guide Fe atom from fh-PHD2 active site lumen to outside. While the mentioned aspartate residue is not free to relay Fe ion movement in f-PHD2 therefore the Fe-rooted repulsion between strand H and HD increases. Such Fe residence in f-PHD2 structure possibly prepares conditions for starting protein assembly and seeding the aggregation.

To estimate the total stability of each PHD2 species, we need a structural criterion to derive protein stability from a single temperature protein unfolding trajectory. To derive such a criterion, we return to the basic definition of the transition temperature (Tm), where the populations of native and unfold structures are in equilibrium.

For a structure *x* residing at *d_x_* along the unfolding pathway, we compute the fraction of structures within 3 angstroms RMSD of *x* with *d* < *d_x_*, and the fraction of structures within 3 angstroms RMSD of *x* with *d* > *d_x_*. The point at which these two fractions become equal represents the transition point and its *d* value (Figure 7). The junction in this graph represents the position of the main transition point of PHD2 species thermal unfolding. We set the cutoff of pairwise RMSD to 3 angstroms because the average RMSD value at *d* = 0.5 with respect to the native state is 3 angstroms. At *d = 0.5* the PHD2 structures are not completely folded nor completely unfolded, and hence do not have a bias toward either state.

The junction point appears in *d* = 0.6 for a-PHD2, while the transition points of f-PHD2 and fh-PHD2 appear at *d* = 0.72 and 0.70 respectively. Therefore, the coordination of Fe (II) to PHD2 structure improves the protein stability, but at the same time prominently enhances the protein aggregation propensity in response to partial unfolding. Possibly, the coordination of the Fe (II) ion to PHD2 stabilizes the states that are reactive for aggregation.

### Conclusion

We conducted MD simulations of the partial thermal unfolding pathways of three species of the PHD2 protein. We clustered structures along the MD trajectories and characterized their corresponding unfolded states.

Partial unfolding is a critical step for protein misfolding and aggregation. We inferred that immature PHD2 (a-PHD2) may aggregate at the last stages of unfolding. However, the functional form of PHD2 is aggregation prone even in the first stages of unfolding.

The addition of the Fe (II) and protons to PHD2 structure severely changes its unfolding pathway states. Among the PHD2 species, f-PHD2 unfolding states may be more susceptible for misfolding and aggregation. Such misfolding propensity arises from positive Δ*G(h)*, high content of strand structures as aggregation triggering structures, Fe ion derived rupture of structure and high exposed hydrophobic area during unfolding. It may provide explanation for PHD2 malfunctions in some normoxic tumor cells.

## Supporting Information

Figure S1 **The interaction energy between Fe ion and histidine residue 374 is indicated.**
(TIF)

Figure S2 **The interaction energy between Fe ion and acidic residues is indicated.**
(TIF)

Figure S3 **It is a scheme of Fe detaching consequence.** Fe atom is pushed out from active site lumen of fh-PHD2 without rupturing active site floor. In f-PHD2, Fe ion tries to escape from active site while D315 traps Fe so it rupture active site floor.
(TIF)

Figure S4 **The animated GIF file shows the fate of Fe atom upon f-PHD2 unfolding.** The Fe atom is mentioned by yellow sphere. The red surface represents active site lumen.
(GIF)

Figure S5 **The animated GIF file shows the fate of Fe atom upon fh-PHD2 unfolding.** The Fe atom is mentioned by yellow sphere. The red surface represents active site lumen.
(GIF)

Table S1 **The metrics used to construct the** ***d*** **metric is represented for a-PHD2.**
(XLS)

Table S2 **The metrics used to construct the** ***d*** **metric is represented for f-PHD2.**
(XLS)

Table S3 **The metrics used to construct the** ***d*** **metric is represented for fh-PHD2.**
(XLS)

Table S4 **The details of structural properties for the states that determined by using affinity propagation clustering, tabulated.** The reported values are the average of the fraction of unfolding that computed for the state’s members.
(DOC)

Table S5 **The details of structural properties for the states that determined by using affinity propagation clustering, tabulated.** The reported values are the average of properties (real observed values) for the state’s members.
(DOC)

Table S6 **The standard error of mean for the average values that reported in Table S5.**
(DOC)
