# Correction: Thermal Unfolding Pathway of PHD2 Catalytic Domain in Three Different PHD2 Species: Computational Approaches

**DOI:** 10.1371/annotation/c414c3ec-b64e-4cf4-bff2-7bb7b3abb558

**Published:** 2013-04-22

**Authors:** Hamid Hadi-Alijanvand, Elizabeth A. Proctor, Bahram Goliaei, Nikolay V. Dokholyan, Ali A. Moosavi-Movahedi

Author Ali A. Moosavi-Movahedi has the wrong affiliations.

The correction institutions he is affiliated with are: 

1) Institute of Biochemistry and Biophysics, University of Tehran, Tehran, Iran

5) Center of Excellence in Biothermodynamics, Institute of Biochemistry and Biophysics, University of Tehran, Tehran, Iran 

