# Supplementary figures and images for: Thermal Unfolding Pathway of PHD2 Catalytic Domain in Three Different PHD2 Species: Computational Approaches
Source: PLoS One. 2012 Oct 15;7(10):e47061. doi: 10.1371/journal.pone.0047061 (PMC3471951; doi:10.1371/journal.pone.0047061)

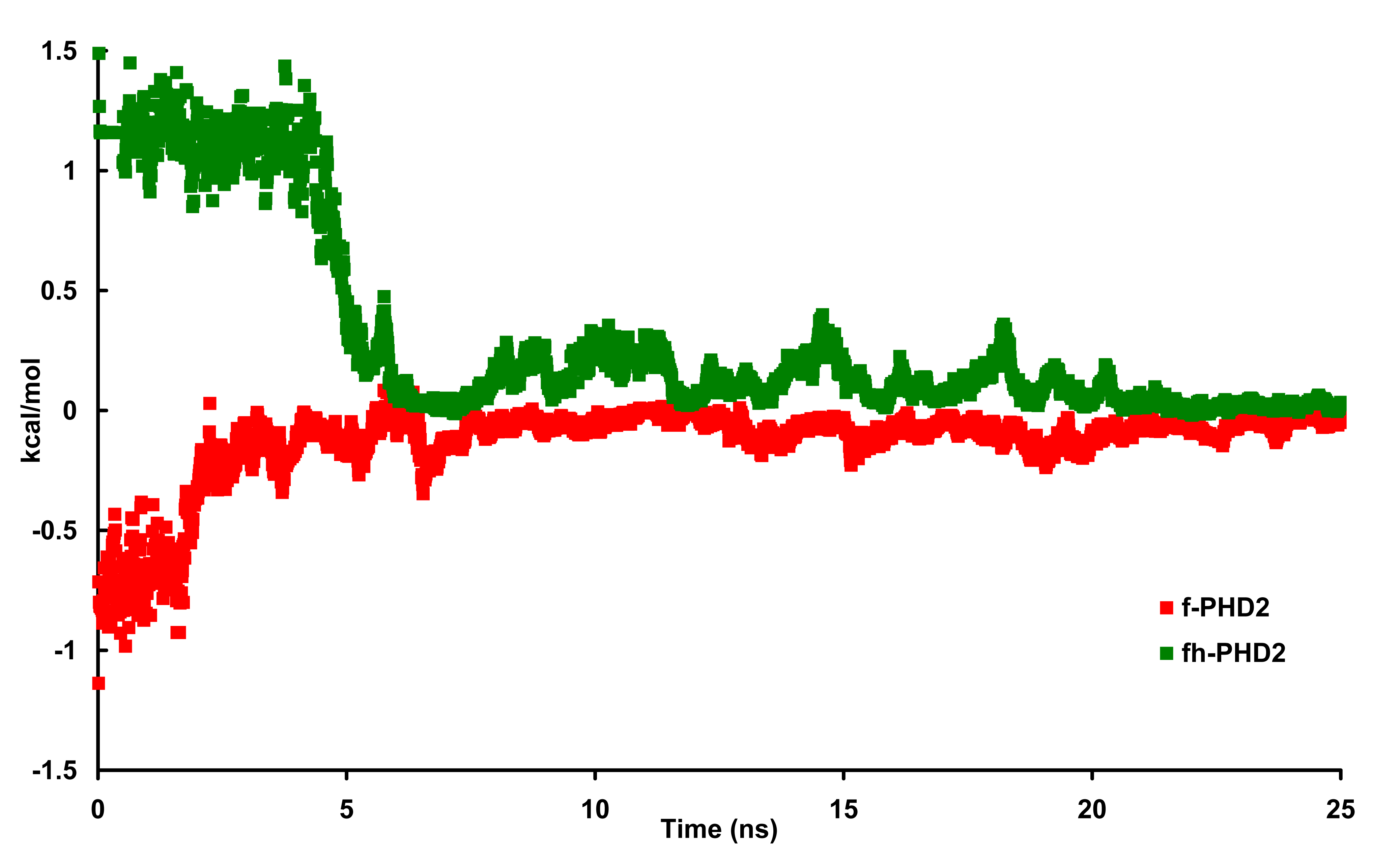

Supplement: Figure S1 — The interaction energy between Fe ion and histidine residue 374 is indicated. (TIF) [file pone.0047061.s001.tif]

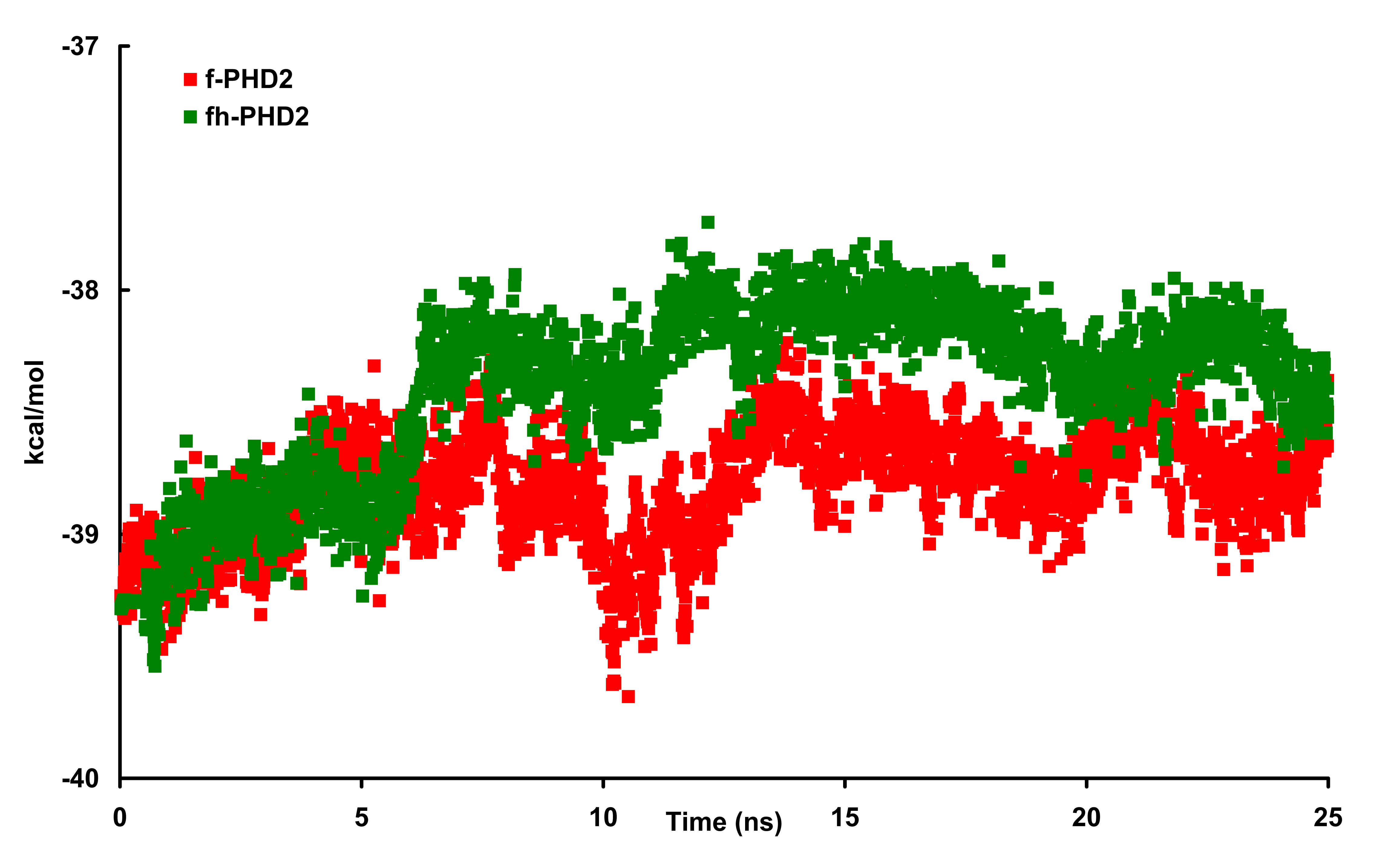

Supplement: Figure S2 — The interaction energy between Fe ion and acidic residues is indicated. (TIF) [file pone.0047061.s002.tif]

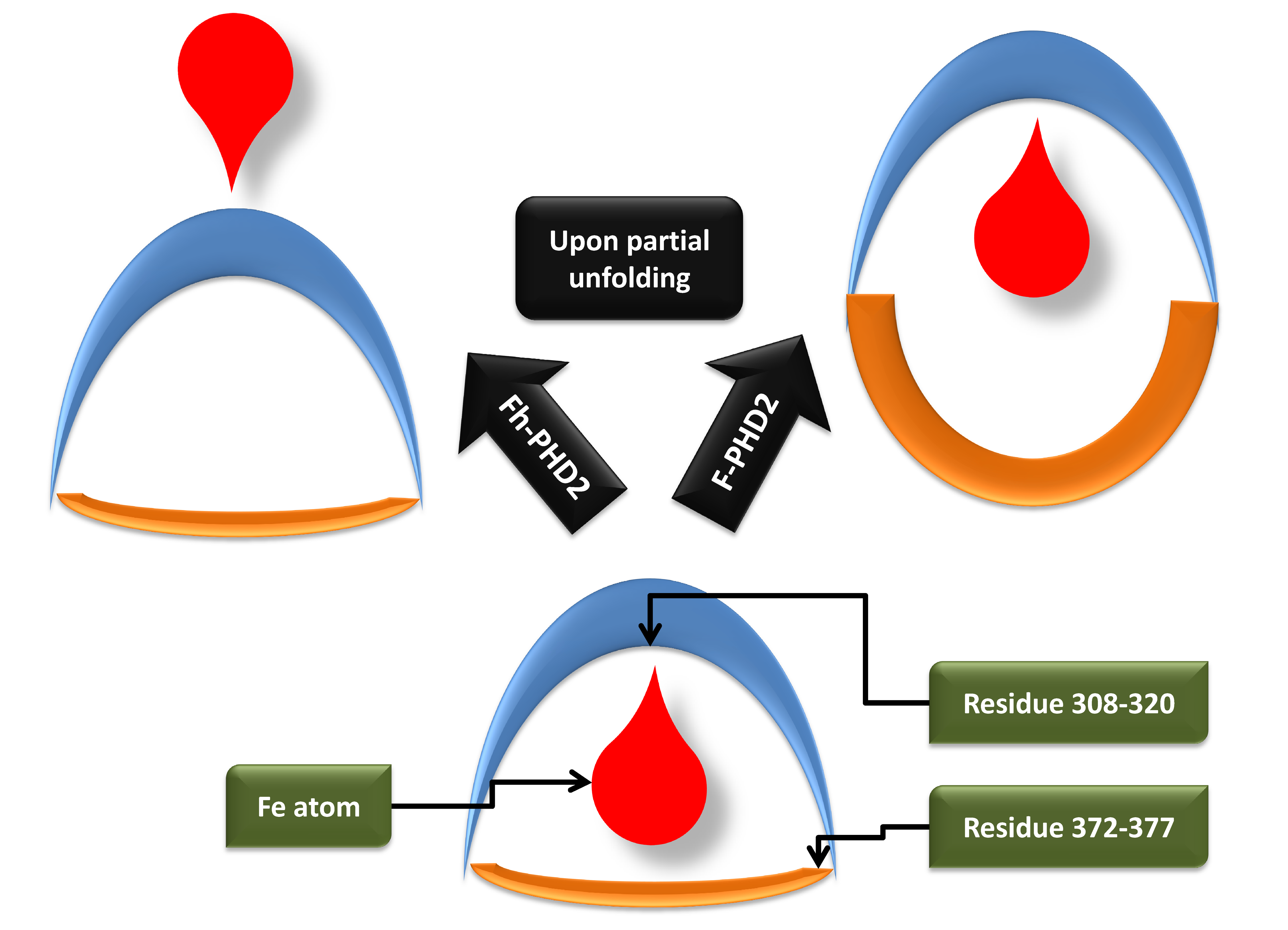

Supplement: Figure S3 — It is a scheme of Fe detaching consequence. Fe atom is pushed out from active site lumen of fh-PHD2 without rupturing active site floor. In f-PHD2, Fe ion tries to escape from active site while D315 traps Fe so it rupture active site floor. (TIF) [file pone.0047061.s003.tif]

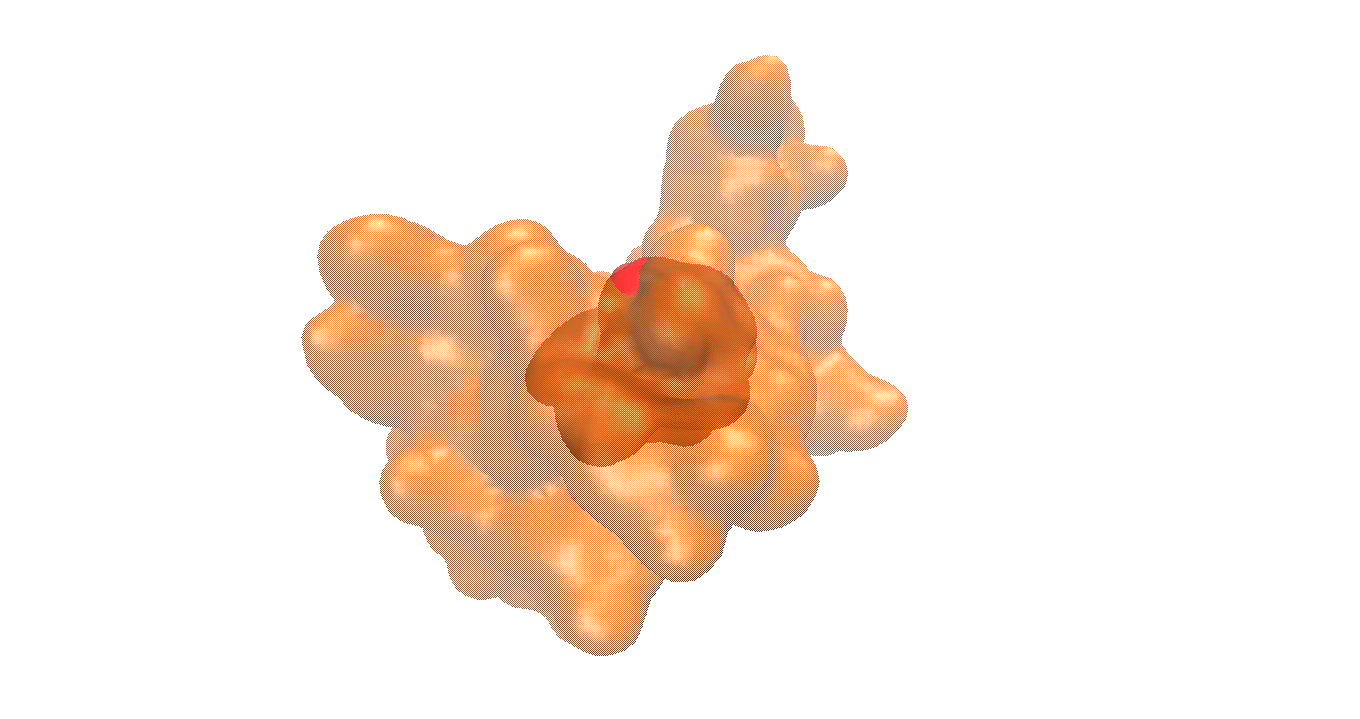

Supplement: Figure S4 — The animated GIF file shows the fate of Fe atom upon f-PHD2 unfolding. The Fe atom is mentioned by yellow sphere. The red surface represents active site lumen. (GIF) [file pone.0047061.s004.gif]

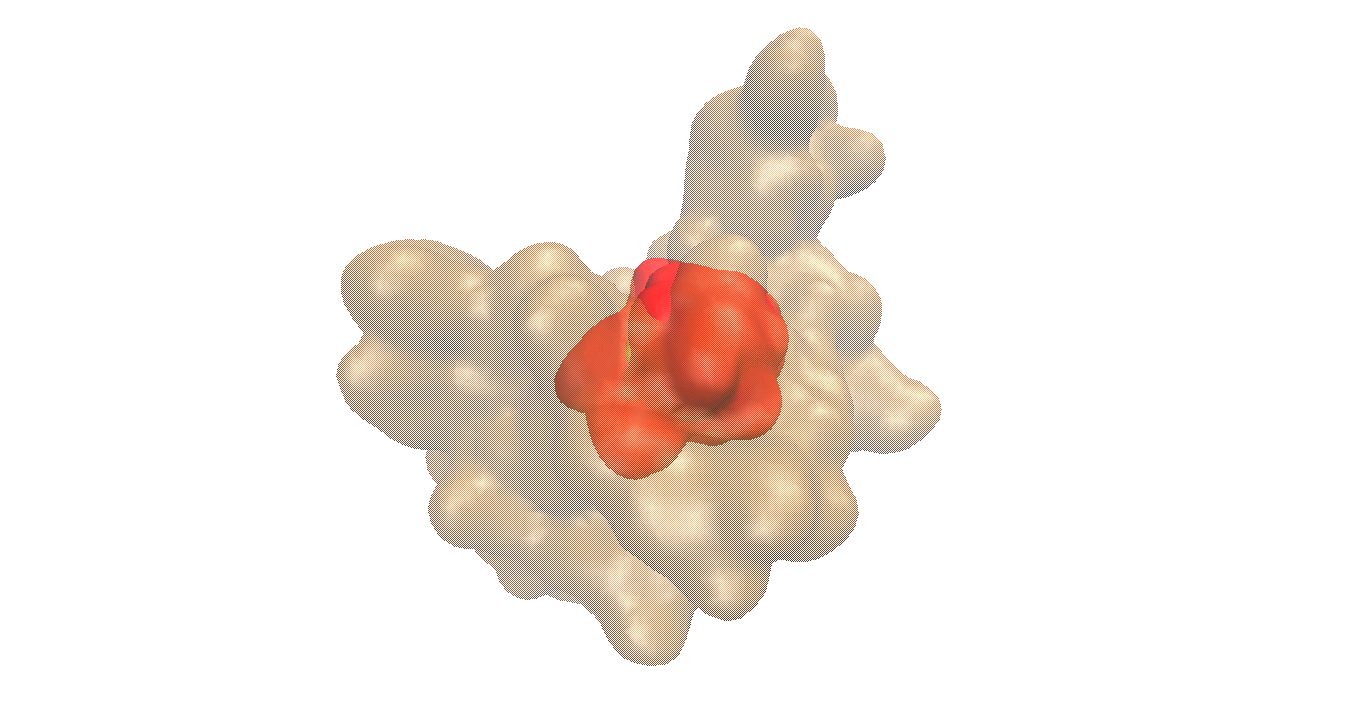

Supplement: Figure S5 — The animated GIF file shows the fate of Fe atom upon fh-PHD2 unfolding. The Fe atom is mentioned by yellow sphere. The red surface represents active site lumen. (GIF) [file pone.0047061.s005.gif]
