# Supplementary material for: Thermal Unfolding Pathway of PHD2 Catalytic Domain in Three Different PHD2 Species: Computational Approaches
Source: PLoS One. 2012 Oct 15;7(10):e47061. doi: 10.1371/journal.pone.0047061 (PMC3471951; doi:10.1371/journal.pone.0047061)
Supplement: Table S4 — The details of structural properties for the states that determined by using affinity propagation clustering, tabulated. The reported values are the average of the fraction of unfolding that computed for the state’s members. (DOC) [file pone.0047061.s009.doc]

Table S4. The details of structural properties for the states that determined by using affinity propagation clustering, tabulated. The reported values are the average of the fraction of unfolding that computed for the state’s members.

| **a-PHD2** |  | Qsh | t.ASA | s.ASA | t.W.ASA | s.np.ASA | s.p.ASA | t.np.ASA | t.p.ASA | s.W258.ASA | s.W334.ASA | s.W367.ASA | s.W389.ASA | ACS-lumen | Docking-site | RMSD | Rgyr | dRMS | ΔCp | Dpm | l.E% | l.H% | l.C% |
| --- | --- | --- | --- | --- | --- | --- | --- | --- | --- | --- | --- | --- | --- | --- | --- | --- | --- | --- | --- | --- | --- | --- | --- |
| Unfolding state | A | 1.00 | 0.00 | 0.00 | 0.00 | 0.00 | 0.00 | 0.00 | 0.00 | 0.00 | 0.00 | 0.00 | 0.00 | 0.00 | 0.00 | 0.00 | 0.00 | 0.00 | 0.00 | 0.00 | 1.00 | 1.00 | 0.00 |
|  | B | 0.60 | 0.28 | 0.30 | 0.13 | 0.25 | 0.34 | 0.25 | 0.29 | 0.63 | 0.03 | 0.04 | 0.21 | 0.26 | 0.26 | 0.24 | 0.30 | 0.26 | 0.10 | 0.33 | 0.98 | 0.82 | 0.11 |
|  | C | 0.44 | 0.46 | 0.49 | 0.30 | 0.43 | 0.54 | 0.43 | 0.49 | 0.92 | 0.11 | 0.11 | 0.42 | 0.50 | 0.46 | 0.42 | 0.44 | 0.41 | 0.24 | 0.76 | 0.75 | 0.63 | 0.34 |
|  | D | 0.20 | 0.73 | 0.73 | 0.57 | 0.68 | 0.77 | 0.71 | 0.75 | 0.74 | 0.24 | 0.76 | 0.38 | 0.55 | 0.63 | 0.65 | 0.66 | 0.69 | 0.55 | 1.00 | 0.40 | 0.23 | 0.63 |
|  | E | 0.09 | 0.87 | 0.85 | 0.66 | 0.74 | 0.95 | 0.76 | 0.96 | 0.51 | 0.26 | 0.96 | 0.62 | 0.83 | 0.69 | 0.83 | 0.84 | 0.86 | 0.07 | 0.68 | 0.30 | 0.04 | 0.76 |
|  | F | 0.00 | 1.00 | 1.00 | 1.00 | 1.00 | 1.00 | 1.00 | 1.00 | 1.00 | 1.00 | 1.00 | 0.82 | 1.00 | 1.00 | 1.00 | 1.00 | 0.99 | 1.00 | 0.96 | 0.00 | 0.05 | 0.96 |
|  | G | 0.03 | 0.95 | 0.95 | 0.83 | 0.91 | 0.99 | 0.92 | 0.98 | 0.79 | 0.47 | 0.83 | 1.00 | 0.93 | 0.88 | 0.99 | 0.97 | 1.00 | 0.71 | 0.58 | 0.05 | 0.00 | 1.00 |
| **f-PHD2** |  | Qsh | t.ASA | s.ASA | t.W.ASA | s.np.ASA | s.p.ASA | t.np.ASA | t.p.ASA | s.W258.ASA | s.W334.ASA | s.W367.ASA | s.W389.ASA | ACS-lumen | Docking-site | RMSD | Rgyr | dRMS | ΔCp | Dpm | l.E% | l.H% | l.C% |
| Unfolding state | A | 1.00 | 0.00 | 0.00 | 0.00 | 0.00 | 0.00 | 0.00 | 0.00 | 0.18 | 0.00 | 0.00 | 0.00 | 0.00 | 0.00 | 0.00 | 0.00 | 0.00 | 0.00 | 0.16 | 1.00 | 1.00 | 0.00 |
|  | B | 0.53 | 0.34 | 0.35 | 0.42 | 0.34 | 0.35 | 0.33 | 0.35 | 0.36 | 0.30 | 0.09 | 0.43 | 0.32 | 0.44 | 0.26 | 0.35 | 0.28 | 0.26 | 0.00 | 0.85 | 0.74 | 0.31 |
|  | C | 0.37 | 0.55 | 0.55 | 0.53 | 0.54 | 0.56 | 0.55 | 0.54 | 0.47 | 0.24 | 0.36 | 0.54 | 0.38 | 0.55 | 0.43 | 0.52 | 0.44 | 0.62 | 0.15 | 0.74 | 0.45 | 0.42 |
|  | D | 0.26 | 0.74 | 0.73 | 0.87 | 0.70 | 0.75 | 0.70 | 0.76 | 1.00 | 0.25 | 0.61 | 1.00 | 0.64 | 0.69 | 0.62 | 0.67 | 0.61 | 0.49 | 0.27 | 0.61 | 0.24 | 0.58 |
|  | E | 0.13 | 0.89 | 0.90 | 0.64 | 0.86 | 0.93 | 0.84 | 0.92 | 0.55 | 0.46 | 0.62 | 0.66 | 0.75 | 0.76 | 0.81 | 0.84 | 0.82 | 0.57 | 0.41 | 0.44 | 0.00 | 0.61 |
|  | F | 0.00 | 0.97 | 0.96 | 0.90 | 0.94 | 0.98 | 0.95 | 0.98 | 0.00 | 1.00 | 1.00 | 0.45 | 1.00 | 1.00 | 0.97 | 0.98 | 0.98 | 0.85 | 1.00 | 0.05 | 0.08 | 0.99 |
|  | G | 0.00 | 1.00 | 1.00 | 1.00 | 1.00 | 1.00 | 1.00 | 1.00 | 0.51 | 0.69 | 0.95 | 0.70 | 0.99 | 0.96 | 1.00 | 1.00 | 1.00 | 1.00 | 0.90 | 0.00 | 0.07 | 1.00 |
| **fh-PHD2** |  | Qsh | t.ASA | s.ASA | t.W.ASA | s.np.ASA | s.p.ASA | t.np.ASA | t.p.ASA | s.W258.ASA | s.W334.ASA | s.W367.ASA | s.W389.ASA | ACS-lumen | Docking-site | RMSD | Rgyr | dRMS | ΔCp | Dpm | l.E% | l.H% | l.C% |
| Unfolding state | A | 1.00 | 0.00 | 0.00 | 0.00 | 0.00 | 0.00 | 0.00 | 0.00 | 0.00 | 0.00 | 0.00 | 0.00 | 0.00 | 0.00 | 0.00 | 0.00 | 0.00 | 0.26 | 0.55 | 1.00 | 1.00 | 0.00 |
|  | B | 0.60 | 0.28 | 0.29 | 0.19 | 0.23 | 0.34 | 0.21 | 0.33 | 0.31 | 0.10 | 0.10 | 0.25 | 0.22 | 0.40 | 0.19 | 0.30 | 0.23 | 0.00 | 0.69 | 0.84 | 0.70 | 0.23 |
|  | C | 0.43 | 0.46 | 0.48 | 0.29 | 0.43 | 0.53 | 0.39 | 0.52 | 0.28 | 0.29 | 0.22 | 0.52 | 0.37 | 0.57 | 0.33 | 0.42 | 0.37 | 0.10 | 0.00 | 0.73 | 0.51 | 0.37 |
|  | D | 0.26 | 0.65 | 0.68 | 0.53 | 0.61 | 0.73 | 0.56 | 0.71 | 0.37 | 0.87 | 0.41 | 0.81 | 0.54 | 0.73 | 0.49 | 0.55 | 0.52 | 0.17 | 0.11 | 0.52 | 0.46 | 0.54 |
|  | E | 0.17 | 0.81 | 0.83 | 0.51 | 0.77 | 0.88 | 0.72 | 0.87 | 0.34 | 0.69 | 0.56 | 0.55 | 0.73 | 1.00 | 0.66 | 0.69 | 0.67 | 0.29 | 1.00 | 0.33 | 0.01 | 0.81 |
|  | F | 0.06 | 0.91 | 0.92 | 0.54 | 0.87 | 0.95 | 0.84 | 0.96 | 0.16 | 0.42 | 0.72 | 0.35 | 0.80 | 0.97 | 0.81 | 0.86 | 0.85 | 0.45 | 0.78 | 0.07 | 0.00 | 0.99 |
|  | G | 0.01 | 0.95 | 0.94 | 0.74 | 0.93 | 0.95 | 0.91 | 0.98 | 0.47 | 0.84 | 0.84 | 0.52 | 0.83 | 0.96 | 0.94 | 0.93 | 0.95 | 0.68 | 0.17 | 0.02 | 0.10 | 1.00 |
|  | H | 0.00 | 1.00 | 1.00 | 1.00 | 1.00 | 1.00 | 1.00 | 1.00 | 1.00 | 1.00 | 1.00 | 1.00 | 1.00 | 0.74 | 1.00 | 1.00 | 1.00 | 1.00 | 0.33 | 0.00 | 0.14 | 0.88 |

The definition of parameters that note here illustrate in article main text. ASA stands for accessible surface area, t, s stands for total and side chain respectively. Rgyr stands for radius of gyration and Dpm notes the dipole moment. The reported values for each state are the average of state members’ value. A is the most fold state and G (H in fh-PHD2) is the most unfold state.
