# Supplementary material for: Thermal Unfolding Pathway of PHD2 Catalytic Domain in Three Different PHD2 Species: Computational Approaches
Source: PLoS One. 2012 Oct 15;7(10):e47061. doi: 10.1371/journal.pone.0047061 (PMC3471951; doi:10.1371/journal.pone.0047061)
Supplement: Table S5 — The details of structural properties for the states that determined by using affinity propagation clustering, tabulated. The reported values are the average of properties (real observed values) for the state’s members. (DOC) [file pone.0047061.s010.doc]

| **a-PHD2** |  | Qsh | t.ASA | s.ASA | t.W.ASA | s.np.ASA | s.p.ASA | t.np.ASA | t.p.ASA | s.W258.ASA | s.W334.ASA | s.W367.ASA | s.W389.ASA | ACS-lumen | Docking-site | RMSD | Rgyr | dRMS | ΔCp | Dpm | l.E% | l.H% | l.C% |
| --- | --- | --- | --- | --- | --- | --- | --- | --- | --- | --- | --- | --- | --- | --- | --- | --- | --- | --- | --- | --- | --- | --- | --- |
| Un folding state | A | 0.8 | 11969.2 | 9271.4 | 195.6 | 2154.6 | 7116.8 | 2855.4 | 9113.8 | 99.4 | 9.3 | 12.5 | 31.3 | 310.6 | 745.3 | 1.2 | 17.6 | 0.3 | -104.7 | 425.3 | 0.0 | -0.1 | 0.3 |
|  | B | 0.6 | 13562.1 | 10618.2 | 225.4 | 2674.3 | 7943.9 | 3474.0 | 10088.1 | 124.7 | 11.3 | 15.1 | 40.6 | 419.4 | 851.9 | 1.9 | 18.0 | 0.6 | -79.6 | 467.1 | 0.0 | -0.1 | 0.4 |
|  | C | 0.6 | 14642.8 | 11474.6 | 266.3 | 3052.1 | 8422.4 | 3920.9 | 10721.9 | 136.0 | 16.7 | 20.7 | 50.6 | 517.1 | 935.8 | 2.5 | 18.2 | 0.7 | -43.3 | 520.0 | -0.1 | -0.2 | 0.7 |
|  | D | 0.4 | 16213.9 | 12554.0 | 329.3 | 3574.4 | 8979.5 | 4609.0 | 11604.9 | 128.9 | 25.7 | 69.2 | 48.5 | 537.8 | 1005.7 | 3.2 | 18.5 | 0.9 | 36.7 | 550.4 | -0.3 | -0.4 | 0.9 |
|  | E | 0.4 | 17030.0 | 13115.4 | 352.3 | 3700.3 | 9415.1 | 4736.0 | 12294.0 | 119.6 | 27.2 | 83.5 | 59.4 | 656.5 | 1031.7 | 3.7 | 18.8 | 1.1 | -85.3 | 510.9 | -0.3 | -0.5 | 1.1 |
|  | F | 0.3 | 17759.1 | 13774.9 | 431.3 | 4246.7 | 9528.2 | 5340.7 | 12418.4 | 139.3 | 78.7 | 86.8 | 68.4 | 725.7 | 1157.9 | 4.2 | 19.0 | 1.2 | 154.4 | 544.9 | -0.4 | -0.5 | 1.2 |
|  | G | 0.4 | 17489.7 | 13568.8 | 391.7 | 4067.3 | 9501.5 | 5134.5 | 12355.2 | 131.1 | 41.6 | 74.1 | 76.7 | 695.9 | 1107.9 | 4.2 | 19.0 | 1.2 | 78.1 | 498.3 | -0.4 | -0.5 | 1.3 |
| **f-PHD2** |  | Qsh | t.ASA | s.ASA | t.W.ASA | s.np.ASA | s.p.ASA | t.np.ASA | t.p.ASA | s.W258.ASA | s.W334.ASA | s.W367.ASA | s.W389.ASA | ACS-lumen | Docking-site | RMSD | Rgyr | dRMS | ΔCp | Dpm | l.E% | l.H% | l.C% |
| Un folding state | A | 0.7 | 12757.5 | 9955.2 | 227.5 | 2384.4 | 7570.7 | 3125.3 | 9607.5 | 107.8 | 13.1 | 23.8 | 30.6 | 335.9 | 786.0 | 1.3 | 17.8 | 0.4 | -90.3 | 429.8 | 0.0 | -0.2 | 0.2 |
|  | B | 0.5 | 14750.7 | 11500.2 | 288.1 | 3089.7 | 8410.5 | 3943.6 | 10771.1 | 114.7 | 29.8 | 29.4 | 49.3 | 465.8 | 911.9 | 2.2 | 18.3 | 0.7 | -24.6 | 393.7 | 0.0 | -0.3 | 0.6 |
|  | C | 0.5 | 15956.9 | 12381.8 | 303.6 | 3483.7 | 8898.1 | 4499.4 | 11389.8 | 119.0 | 26.3 | 47.4 | 54.2 | 490.5 | 943.6 | 2.8 | 18.6 | 0.9 | 64.6 | 428.6 | -0.1 | -0.4 | 0.8 |
|  | D | 0.4 | 17093.0 | 13186.7 | 353.0 | 3828.0 | 9358.7 | 4866.7 | 12152.1 | 139.0 | 27.3 | 63.8 | 74.4 | 599.7 | 981.9 | 3.5 | 18.9 | 1.2 | 31.7 | 457.1 | -0.2 | -0.5 | 1.0 |
|  | E | 0.3 | 17958.3 | 13915.5 | 320.6 | 4142.0 | 9773.5 | 5213.4 | 12669.0 | 122.0 | 38.7 | 64.2 | 59.7 | 642.1 | 1001.2 | 4.2 | 19.1 | 1.4 | 53.3 | 488.5 | -0.3 | -0.6 | 1.0 |
|  | F | 0.3 | 18441.8 | 14203.7 | 358.0 | 4309.8 | 9893.8 | 5490.9 | 12876.6 | 101.0 | 68.8 | 89.2 | 50.2 | 745.0 | 1070.4 | 4.7 | 19.4 | 1.6 | 124.3 | 626.0 | -0.5 | -0.5 | 1.5 |
|  | G | 0.3 | 18611.8 | 14376.5 | 371.9 | 4433.4 | 9943.2 | 5606.5 | 12935.0 | 120.5 | 51.7 | 85.8 | 61.4 | 741.4 | 1059.0 | 4.8 | 19.4 | 1.6 | 161.0 | 602.1 | -0.6 | -0.5 | 1.5 |
| **fh-PHD2** |  | Qsh | t.ASA | s.ASA | t.W.ASA | s.np.ASA | s.p.ASA | t.np.ASA | t.p.ASA | s.W258.ASA | s.W334.ASA | s.W367.ASA | s.W389.ASA | ACS-lumen | Docking-site | RMSD | Rgyr | dRMS | ΔCp | Dpm | l.E% | l.H% | l.C% |
| Un folding state | A | 0.9 | 11663.2 | 9049.3 | 189.0 | 2101.6 | 6905.8 | 2825.4 | 8795.9 | 101.2 | 7.0 | 13.8 | 21.2 | 278.3 | 741.1 | 0.9 | 17.5 | 0.2 | -14.3 | 505.7 | 0.1 | -0.1 | 0.2 |
|  | B | 0.7 | 13443.7 | 10506.1 | 246.5 | 2594.7 | 7868.0 | 3373.9 | 10026.4 | 112.1 | 9.3 | 28.9 | 38.5 | 371.2 | 832.1 | 1.7 | 18.0 | 0.5 | -87.4 | 517.2 | 0.0 | -0.2 | 0.4 |
|  | C | 0.6 | 14621.1 | 11474.0 | 278.1 | 3033.6 | 8383.4 | 3841.6 | 10722.5 | 111.1 | 13.6 | 45.5 | 57.0 | 434.7 | 868.4 | 2.2 | 18.3 | 0.7 | -57.9 | 462.2 | -0.1 | -0.3 | 0.6 |
|  | D | 0.5 | 15849.6 | 12454.1 | 350.8 | 3419.7 | 8956.3 | 4308.9 | 11462.6 | 114.2 | 26.4 | 73.1 | 76.5 | 508.9 | 905.0 | 2.8 | 18.5 | 0.9 | -40.0 | 471.2 | -0.2 | -0.3 | 0.8 |
|  | E | 0.4 | 16831.9 | 13224.9 | 344.8 | 3780.7 | 9360.8 | 4717.3 | 12031.2 | 113.2 | 22.4 | 95.0 | 59.0 | 590.1 | 966.3 | 3.4 | 18.8 | 1.1 | -4.1 | 541.6 | -0.3 | -0.5 | 1.0 |
|  | F | 0.4 | 17520.8 | 13659.4 | 353.5 | 3998.8 | 9575.2 | 5031.2 | 12404.2 | 107.0 | 16.4 | 118.1 | 45.1 | 621.9 | 958.6 | 4.0 | 19.1 | 1.3 | 40.2 | 524.1 | -0.4 | -0.5 | 1.2 |
|  | G | 0.3 | 17777.2 | 13763.8 | 413.0 | 4117.8 | 9561.3 | 5218.5 | 12473.9 | 117.7 | 25.9 | 136.6 | 56.5 | 635.4 | 957.8 | 4.5 | 19.2 | 1.5 | 106.3 | 476.0 | -0.4 | -0.4 | 1.2 |
|  | H | 0.3 | 18074.5 | 14058.9 | 493.2 | 4273.0 | 9702.7 | 5455.8 | 12535.5 | 136.3 | 29.4 | 159.1 | 89.6 | 706.3 | 908.6 | 4.8 | 19.4 | 1.5 | 197.1 | 488.6 | -0.4 | -0.4 | 1.1 |

Table S5. The details of structural properties for the states that determined by using affinity propagation clustering, tabulated. The reported values are the average of properties for the state’s members.
