# Supplementary material for: Thermal Unfolding Pathway of PHD2 Catalytic Domain in Three Different PHD2 Species: Computational Approaches
Source: PLoS One. 2012 Oct 15;7(10):e47061. doi: 10.1371/journal.pone.0047061 (PMC3471951; doi:10.1371/journal.pone.0047061)
Supplement: Table S6 — The standard error of mean for the average values that reported in Table S5. (DOC) [file pone.0047061.s011.doc]

Table S6. The standard error of mean for the average values that reported in Table S5.

| **a-PHD2** |  | Qsh | t.ASA | s.ASA | t.W.ASA | s.np.ASA | s.p.ASA | t.np.ASA | t.p.ASA | s.W258.ASA | s.W334.ASA | s.W367.ASA | s.W389.ASA | ACS-lumen | Docking-site | RMSD | Rgyr | dRMS | ΔCp | Dpm | l.E% | l.H% | l.C% |
| --- | --- | --- | --- | --- | --- | --- | --- | --- | --- | --- | --- | --- | --- | --- | --- | --- | --- | --- | --- | --- | --- | --- | --- |
| Un folding state | A | 0.009 | 59.30 | 50.80 | 1.14 | 14.42 | 36.74 | 14.99 | 44.77 | 0.63 | 0.30 | 0.32 | 0.85 | 6.57 | 2.72 | 0.03 | 0.02 | 0.01 | 5.52 | 2.10 | 0.01 | 0.01 | 0.02 |
|  | B | 0.003 | 28.56 | 23.52 | 1.51 | 8.08 | 16.83 | 10.54 | 19.42 | 1.31 | 0.38 | 0.44 | 0.56 | 3.09 | 2.79 | 0.02 | 0.01 | 0.00 | 3.11 | 3.48 | 0.01 | 0.01 | 0.01 |
|  | C | 0.002 | 23.67 | 19.18 | 1.80 | 8.66 | 12.38 | 10.42 | 14.91 | 1.26 | 0.31 | 0.53 | 1.08 | 2.81 | 2.44 | 0.01 | 0.00 | 0.00 | 3.18 | 2.04 | 0.01 | 0.01 | 0.01 |
|  | D | 0.001 | 17.22 | 11.95 | 1.43 | 5.66 | 7.57 | 6.75 | 11.71 | 0.80 | 0.36 | 0.61 | 1.03 | 2.23 | 2.89 | 0.01 | 0.00 | 0.00 | 2.28 | 1.13 | 0.00 | 0.01 | 0.01 |
|  | E | 0.001 | 11.83 | 9.67 | 1.15 | 4.54 | 6.90 | 5.13 | 8.95 | 0.68 | 0.38 | 0.74 | 0.47 | 2.54 | 2.76 | 0.01 | 0.01 | 0.00 | 2.62 | 2.10 | 0.00 | 0.00 | 0.01 |
|  | F | 0.001 | 15.19 | 11.41 | 2.10 | 6.19 | 8.01 | 7.70 | 12.80 | 1.26 | 1.27 | 1.21 | 0.91 | 2.71 | 4.24 | 0.00 | 0.00 | 0.00 | 4.71 | 2.72 | 0.00 | 0.01 | 0.01 |
|  | G | 0.001 | 15.88 | 13.88 | 1.46 | 6.66 | 9.23 | 7.19 | 11.88 | 0.85 | 0.51 | 0.86 | 0.53 | 2.49 | 3.46 | 0.02 | 0.01 | 0.00 | 3.61 | 1.73 | 0.00 | 0.01 | 0.01 |
| **f-PHD2** |  | Qsh | t.ASA | s.ASA | t.W.ASA | s.np.ASA | s.p.ASA | t.np.ASA | t.p.ASA | s.W258.ASA | s.W334.ASA | s.W367.ASA | s.W389.ASA | ACS-lumen | Docking-site | RMSD | Rgyr | dRMS | ΔCp | Dpm | l.E% | l.H% | l.C% |
| Un folding state | A | 0.009 | 67.84 | 57.40 | 2.55 | 17.05 | 41.21 | 19.55 | 49.10 | 1.12 | 0.34 | 0.77 | 0.61 | 4.20 | 3.63 | 0.03 | 0.02 | 0.01 | 5.26 | 2.69 | 0.01 | 0.01 | 0.02 |
|  | B | 0.002 | 25.24 | 18.24 | 1.35 | 10.32 | 9.13 | 12.36 | 13.21 | 0.57 | 0.57 | 0.66 | 0.50 | 1.97 | 3.06 | 0.01 | 0.01 | 0.00 | 3.43 | 2.38 | 0.01 | 0.01 | 0.01 |
|  | C | 0.001 | 21.14 | 16.71 | 1.72 | 7.28 | 13.13 | 9.56 | 14.66 | 0.85 | 0.45 | 0.76 | 0.57 | 2.44 | 3.17 | 0.01 | 0.00 | 0.00 | 4.13 | 2.45 | 0.01 | 0.01 | 0.01 |
|  | D | 0.001 | 19.20 | 16.38 | 1.36 | 6.58 | 11.00 | 6.43 | 14.03 | 0.65 | 0.43 | 0.77 | 0.55 | 2.07 | 2.37 | 0.01 | 0.00 | 0.00 | 2.63 | 2.11 | 0.00 | 0.01 | 0.01 |
|  | E | 0.001 | 12.11 | 10.92 | 1.41 | 6.60 | 6.54 | 7.37 | 7.54 | 0.87 | 0.63 | 0.68 | 0.73 | 2.20 | 1.99 | 0.01 | 0.00 | 0.00 | 3.28 | 1.70 | 0.00 | 0.01 | 0.01 |
|  | F | 0.001 | 14.38 | 10.54 | 1.51 | 7.46 | 6.68 | 9.13 | 8.13 | 0.79 | 0.92 | 0.66 | 0.72 | 3.14 | 2.69 | 0.01 | 0.01 | 0.00 | 3.83 | 3.44 | 0.01 | 0.01 | 0.01 |
|  | G | 0.001 | 11.73 | 10.75 | 1.13 | 7.12 | 6.24 | 6.74 | 6.77 | 0.73 | 0.59 | 0.47 | 0.64 | 2.50 | 2.46 | 0.01 | 0.00 | 0.00 | 2.54 | 2.49 | 0.01 | 0.01 | 0.01 |
| **fh-PHD2** |  | Qsh | t.ASA | s.ASA | t.W.ASA | s.np.ASA | s.p.ASA | t.np.ASA | t.p.ASA | s.W258.ASA | s.W334.ASA | s.W367.ASA | s.W389.ASA | ACS-lumen | Docking-site | RMSD | Rgyr | dRMS | ΔCp | Dpm | l.E% | l.H% | l.C% |
| Un folding state | A | 0.006 | 36.83 | 32.47 | 1.14 | 10.53 | 22.66 | 11.92 | 25.64 | 0.63 | 0.25 | 0.16 | 0.43 | 3.42 | 2.61 | 0.03 | 0.02 | 0.01 | 2.24 | 2.95 | 0.01 | 0.01 | 0.01 |
|  | B | 0.003 | 39.46 | 30.48 | 1.83 | 12.64 | 19.05 | 13.46 | 27.37 | 0.96 | 0.40 | 0.85 | 0.53 | 3.34 | 3.86 | 0.01 | 0.01 | 0.00 | 3.94 | 2.28 | 0.01 | 0.01 | 0.02 |
|  | C | 0.002 | 25.96 | 21.58 | 1.61 | 8.82 | 16.57 | 8.89 | 20.29 | 1.16 | 0.35 | 0.95 | 0.87 | 2.71 | 3.39 | 0.01 | 0.00 | 0.00 | 4.83 | 1.97 | 0.01 | 0.01 | 0.01 |
|  | D | 0.001 | 22.89 | 17.61 | 1.44 | 6.73 | 12.49 | 7.34 | 17.59 | 0.78 | 0.46 | 0.78 | 0.64 | 2.50 | 2.68 | 0.01 | 0.01 | 0.00 | 3.65 | 1.97 | 0.00 | 0.01 | 0.01 |
|  | E | 0.001 | 12.00 | 10.50 | 1.48 | 5.55 | 7.46 | 6.19 | 8.55 | 0.68 | 0.35 | 1.15 | 0.57 | 2.14 | 2.03 | 0.01 | 0.00 | 0.00 | 2.99 | 2.04 | 0.00 | 0.01 | 0.01 |
|  | F | 0.001 | 10.02 | 6.57 | 1.25 | 5.36 | 5.45 | 6.55 | 6.16 | 0.56 | 0.29 | 0.72 | 0.47 | 1.84 | 2.12 | 0.01 | 0.00 | 0.00 | 3.01 | 3.37 | 0.00 | 0.01 | 0.01 |
|  | G | 0.001 | 10.34 | 7.66 | 1.12 | 5.38 | 5.81 | 6.33 | 7.48 | 0.56 | 0.44 | 0.91 | 0.58 | 2.11 | 2.34 | 0.01 | 0.00 | 0.00 | 3.26 | 2.02 | 0.00 | 0.01 | 0.01 |
|  | H | 0.001 | 15.55 | 15.56 | 1.79 | 8.42 | 10.28 | 8.98 | 10.55 | 0.87 | 0.48 | 1.19 | 1.31 | 3.01 | 3.15 | 0.01 | 0.00 | 0.00 | 4.35 | 1.97 | 0.00 | 0.01 | 0.01 |
